# New-Onset Atrial Flutter in Pneumonia: A Systemic Inflammatory Response With Cardiac Implications

**DOI:** 10.7759/cureus.79609

**Published:** 2025-02-25

**Authors:** Muhammad Faraaz Ismail, Nabeelah Ismail, Jamsheer Ambalath

**Affiliations:** 1 General Practice, Thumbay University Hospital, Ajman, ARE; 2 Pulmonology, Thumbay University Hospital, Ajman, ARE

**Keywords:** arrhythmia, atrial flutter, cardiac complications of pneumonia, pneumonia, systemic inflammation

## Abstract

Atrial arrhythmias, particularly atrial flutter, are increasingly recognized as complications of systemic infections, including community-acquired pneumonia (CAP). This report describes the case of a 60-year-old male with CAP complicated by new-onset atrial flutter, highlighting the role of systemic inflammation and electrolyte imbalances in arrhythmogenesis. The systemic inflammatory response associated with pneumonia leads to cytokine-mediated atrial remodeling, autonomic dysfunction, and direct myocardial stress, all of which can contribute to atrial flutter. Furthermore, electrolyte disturbances such as hypokalemia and hyponatremia, both present in this patient, exacerbate cardiac excitability, prolong repolarization, and promote reentry circuits, increasing susceptibility to atrial flutter compared to other arrhythmias. Despite the severity of the arrhythmia, cardioversion was not considered in this case, as the patient remained hemodynamically stable with effective rate control achieved through IV amiodarone. Instead, management focused on targeted interventions, including electrolyte correction, infection control, and anticoagulation therapy, to mitigate thromboembolic risk. Given the well-documented link between systemic inflammation, electrolyte imbalances, and arrhythmias, early monitoring of electrolytes, inflammatory markers, and ECG in pneumonia patients may allow for timely interventions and improved clinical outcomes. Future research should focus on prospective trials evaluating standardized arrhythmia screening and electrolyte correction protocols in pneumonia patients at high risk for cardiovascular complications. Retrospective cohort studies assessing the impact of inflammation-targeted therapies on arrhythmia incidence may also help refine clinical guidelines. Recognizing pneumonia as a multi-system disease rather than an isolated respiratory infection may lead to improved risk stratification and therapeutic strategies, ultimately improving patient outcomes.

## Introduction

Pneumonia remains a leading cause of hospitalization and mortality worldwide, particularly in older adults and those with comorbidities such as diabetes mellitus and cardiovascular disease [1]. The systemic inflammatory response triggered by pneumonia is increasingly recognized as a precipitant for secondary complications, including cardiovascular dysfunction [2]. While both atrial fibrillation (AF) and atrial flutter can occur as complications of pneumonia, AF is the more commonly observed arrhythmia in critically ill patients [3]. New-onset atrial arrhythmias, including AF and atrial flutter, have been reported in 4-10% of hospitalized pneumonia patients and are associated with worse clinical outcomes [3,4].

The mechanisms linking pneumonia to atrial arrhythmias are multifaceted, involving systemic inflammation, hypoxia, autonomic dysregulation, direct myocardial injury, and electrolyte imbalances [2,5]. Elevated inflammatory markers, particularly C-reactive protein (CRP), have been associated with an increased risk of AF and atrial flutter, highlighting inflammation as a key driver of arrhythmogenicity [6]. Cytokines such as interleukin (IL)-6, tumor necrosis factor-alpha (TNF-α), and IL-1β have been implicated in the inflammatory cascade, leading to endothelial dysfunction and myocardial stress, thereby increasing the susceptibility to arrhythmias [7]. Furthermore, the inflammatory burden in pneumonia varies by age, with older adults demonstrating an exaggerated cytokine response, which may contribute to the higher incidence of cardiovascular complications in this population [1].

Beyond inflammation, electrolyte imbalances such as hypokalemia and hyponatremia frequently accompany pneumonia, predisposing patients to atrial arrhythmias [7,8]. While hypokalemia is strongly associated with both AF and atrial flutter due to its effects on atrial conduction and reentry circuits, the role of hyponatremia in atrial flutter is less well established [8]. Potassium disturbances can prolong atrial conduction, increase automaticity, and promote reentry circuits, thereby facilitating the development of AF [5,7]. Diuretic therapy, commonly used to manage pulmonary congestion in pneumonia, may contribute to electrolyte disturbances, increasing the risk of arrhythmias [7].

This report presents the case of a 60-year-old male with CAP complicated by new-onset atrial flutter, illustrating the complex interplay between systemic inflammation, electrolyte imbalances, and cardiac arrhythmias. By examining this case, we emphasize the importance of early recognition and intervention to mitigate the cardiovascular complications associated with pneumonia.

## Case presentation

A 60-year-old male with a history of type 2 diabetes mellitus, hypertension, and previous prostate cancer treated surgically five years ago presented to the emergency department (ED) with complaints of fever, headache, and generalized body pain. He was managed on an outpatient basis and treated symptomatically. The patient was a chronic smoker (30 pack years) and was on metformin 1000 mg twice daily (BD), saxagliptin 5 mg once daily (OD), and candesartan 16 mg OD for his comorbidities. He had no known drug allergies. He was sent home with a combination of paracetamol and ibuprofen, oseltamivir, and azithromycin. 

Four days later, he returned to the ED with complaints of high-grade fever, dyspnea, and fatigue for five days. He reported associated chills and intermittent dry cough. In spite of the initial management from his previous visit, he experienced worsening lethargy and respiratory difficulty on the day of admission.

Upon arrival at the ED at the second presentation, the patient was hemodynamically stable but tachycardic, with an irregular heart rate of 132 beats per minute (reference: 60-100 bpm). His blood pressure was 137/90 mmHg (reference: 90-120/60-80 mmHg), and his respiratory rate was 30 breaths per minute (reference: 12-20/min). His temperature measured 36.6°C and oxygen saturation (SpO₂) was 94% on room air. A random blood glucose test (GRBS) revealed severe hyperglycemia at 515 mg/dL (reference: <140 mg/dL). The absence of high-grade fever on initial presentation could be attributed to antipyretic use by the patient.

While in the ED and after initial evaluation, the patient developed a new-onset atrial flutter with right bundle branch block (RBBB), confirmed by ECG. He had no prior history of AF/atrial flutter or any documented cardiac arrhythmias. The patient was admitted to the ICU for close monitoring and aggressive management due to progressive respiratory distress and new-onset atrial flutter, necessitating prompt intervention for hemodynamic stabilization and infection control. These findings indicated systemic stress and metabolic dysregulation, likely driven by the underlying infectious and inflammatory process. Severe hyperglycemia raised concerns for infection-induced metabolic decompensation, warranting further evaluation to exclude diabetic ketoacidosis (DKA) or hyperosmolar hyperglycemic state (HHS).

Laboratory investigations in ICU

Initial laboratory findings indicated marked systemic inflammation, with a C-reactive protein (CRP) level of 375 mg/L (reference: <10 mg/L), suggesting a significant inflammatory response. Electrolyte imbalances were observed, with sodium at 123.6 mmol/L (reference: 135-145 mmol/L) and potassium at 3.5 mmol/L (reference: 3.5-5.1 mmol/L). Renal function tests (RFTs) revealed elevated urea at 78.5 mg/dL (reference: 7-20 mg/dL), and normal creatinine at 0.99 mg/dL, suggesting prerenal azotemia secondary to dehydration or infection-related metabolic changes. Routine urine analysis revealed significant glucosuria (++++) and moderate ketonuria (++). Arterial blood gas (ABG) analysis showed a pH of 7.38, pO₂ of 66 mmHg (reference: 75-100 mmHg), pCO₂ of 30 mmHg (reference: 35-45 mmHg), and bicarbonate (HCO₃) of 17.7 mmol/L (reference: 22-28 mmol/L), indicating mild metabolic acidosis.

These findings, particularly the absence of severe metabolic acidosis (pH 7.38, HCO₃ 17.7 mmol/L) in spite of ketonuria, along with the lack of a significantly elevated anion gap, effectively ruled out DKA as the primary cause of metabolic decompensation. Instead, the mild metabolic acidosis was more consistent with a systemic inflammatory response and respiratory compromise secondary to pneumonia.

Additional notable findings included a low albumin level of 2.92 g/dL (normal: 3.5-5 g/dL), consistent with a negative acute-phase response. Coagulation studies showed an elevated D-dimer level of 1752 ng/mL (normal: <500 ng/mL), suggesting systemic inflammation and a hypercoagulable state, a slightly prolonged partial thromboplastin time (PTT) of 37.3 sec (normal: 25-35 sec) and an INR of 0.9 (normal: <1.1). A CHA₂DS₂-VASc score for this patient was 2 (hypertension and DM) which indicated a moderate thromboembolic risk, warranting oral anticoagulation therapy as per guideline recommendations. Given the absence of prior thromboembolic events, the anticoagulation strategy was preventive rather than therapeutic

Management of atrial flutter and pneumonia

Rate control for atrial flutter was initiated with IV amiodarone (300 mg bolus, followed by 900 mg over 24 hours). Due to the increased thromboembolic risk associated with atrial flutter, therapeutic anticoagulation was started with subcutaneous enoxaparin 60 mg BD, and later transitioned to oral edoxaban 60 mg OD. Pulmonary congestion, as evidenced by bilateral crepitations on auscultation of the lung bases, was managed with IV furosemide (5 mg/hour continuous infusion), later adjusted to 40 mg IV three times daily (TDS).

Broad-spectrum antimicrobial therapy was initiated with IV meropenem (1 g TDS) and azithromycin (500 mg OD). Oxygen therapy was provided via nasal cannula at 2 L/minute, titrated based on oxygen saturation, with nebulized budesonide and ipratropium bromide for symptom relief. Hypokalemia was managed with IV potassium chloride (KCl) 40 mEq via central access, supplemented with oral KCl syrup. Hyponatremia correction was approached cautiously with strict fluid balance monitoring to prevent rapid overcorrection and osmotic demyelination syndrome.

Hyperglycemia was initially treated with IV regular insulin infusion (5 IU/hr), which was later transitioned to subcutaneous insulin glargine (18 IU at bedtime) and pre-meal insulin aspart adjusted via a sliding scale.

Imaging and other investigations

**Figure 1:**
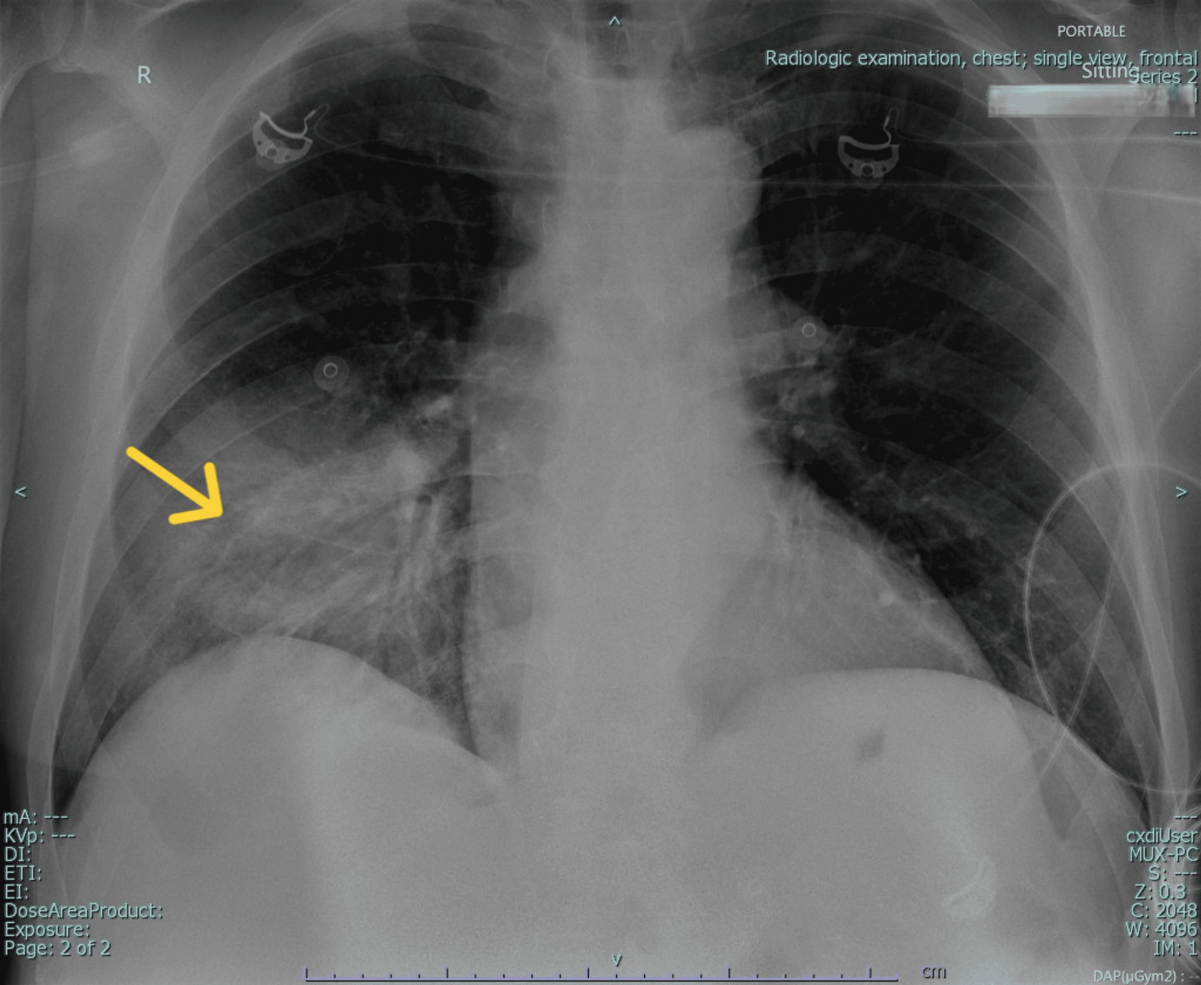
Chest x-ray (portable, anteroposterior view) showing a focal heterogeneous opacity in the right lower lobe, suggestive of focal pneumonia (yellow arrow).

**Figure 2:**
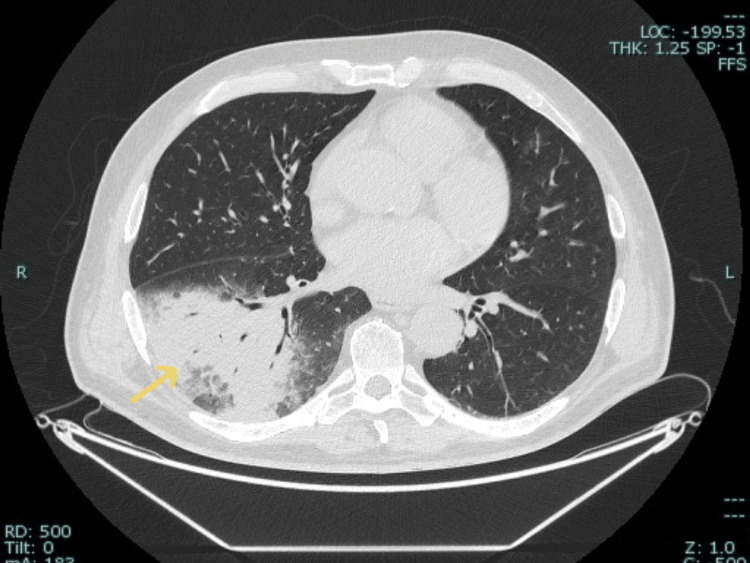
Non-contrast CT scan of the chest showing large consolidation in the right lower lobe, consistent with pneumonia (yellow arrow).

To further evaluate the severity of pneumonia, the patient underwent a portable anteroposterior (AP) chest X-ray (Figure 1) followed by a non-contrast CT scan of the chest (Figure 2). The chest X-ray, performed on the day of the second presentation (Hospital Day 1), revealed bilateral increased bronchovascular markings along with reticular nodular opacities in both lungs, suggestive of the early infectious changes associated with pneumonia. A focal heterogeneous opacity was noted in the right lower lobe, suggestive of focal pneumonia. The bilateral costophrenic angles were clear, with no evidence of pleural effusion or mediastinal shift, and the cardiac silhouette appeared unremarkable. The non-contrast CT of the chest, performed one day later (Hospital Day 2), confirmed a large consolidation in the right lower lobe with air bronchograms and surrounding ground-glass opacities, consistent with infectious pneumonia.

On Hospital day 2, troponin I was also tested to evaluate for potential myocardial stress or subclinical ischemia due to the ongoing systemic inflammatory response and new-onset arrhythmia. The result was negative (<0.16 ng/mL), ruling out acute coronary syndrome as a contributing factor.

Clinical progress

The patient's hemodynamic and respiratory status progressively improved over the course of his ICU stay, with close monitoring and targeted interventions. Arrhythmia control was achieved with amiodarone, and there were no further episodes of tachyarrhythmia following initial stabilization. Inflammatory markers showed a steady downward trend, reflecting a positive response to antimicrobial therapy and the resolution of systemic inflammation. Electrolyte imbalances were successfully corrected, and renal function remained stable throughout hospitalization.

On Hospital Day 3, CRP had decreased to 156 mg/L, indicating a reduction in systemic inflammation. With stable cardiac rhythm, the intravenous amiodarone infusion was discontinued, and the patient was transitioned to oral amiodarone 200 mg OD for continued rate control. Therapeutic anticoagulation with enoxaparin was stopped, and edoxaban 60 mg OD was continued as per standard thromboprophylaxis guidelines for atrial flutter. The patient remained hemodynamically stable, and pulmonary congestion improved (as per auscultatory findings), allowing for a gradual reduction in diuretic therapy. Oxygen supplementation was progressively weaned to 1L/minute and the patient maintained a saturation of 96%.

By Hospital Day 4, the patient demonstrated further clinical improvement, with a CRP decline to 93.5 mg/L, normalized sodium and potassium levels, and an increased tolerance for oral intake. Oxygen supplementation was continued intermittently at 1L/minute. On room air, the patient maintained oxygen saturation levels ≥95%. Blood glucose levels remained well-controlled with subcutaneous insulin glargine (18 IU at bedtime) and prandial insulin aspart per sliding scale adjustments. The patient was encouraged to mobilize, and gradual ambulation was initiated under supervision to assess functional status before discharge.

On Hospital Day 5, CRP further declined to 54.2 mg/L, with resolution of electrolyte imbalances and normalization of renal function parameters. Potassium and sodium correction were effectively achieved, with levels reaching 4.0 mmol/L and 138 mmol/L, respectively. Continued post-discharge monitoring was advised to prevent recurrence. The patient remained clinically stable, afebrile, and normotensive. Oxygen therapy was discontinued, and the patient was maintained on oral medications for ongoing management. Oral amiodarone was planned to be continued, with periodic reassessment for potential tapering. With a CHA₂DS₂-VASc score of 2, edoxaban therapy was continued for stroke prevention. Based on the clinical response of the patient, cardioversion was not considered at this time. However, based on the outcomes of future reassessments, it may still be considered.

With sustained clinical stability, the patient was planned for transition to ward-based care for continued monitoring before discharge. Pre-discharge assessments included evaluating frailty and deconditioning, ensuring the patient’s readiness for discharge. Dietary recommendations emphasized a low-fat, high-protein regimen, with sodium restriction considered in the context of cardiovascular risk​ as well as a diabetic-friendly regimen with adjusted fluid intake. The patient was educated on the importance of medication adherence, lifestyle modifications, and follow-up evaluations to ensure long-term arrhythmia control and cardiovascular risk reduction.

For ease of interpretation, all laboratory values and their respective trends have been tabulated, allowing for clear visualization of the patient’s progression over time (Table 1).

**Table 1:** Laboratory trends of key biomarkers during admission and ICU stay CRP: C-reactive protein; WBC: white blood cells

Date	CRP (mg/L)	WBC (10³/µL)	Sodium (mmol/L)	Potassium (mmol/L)	Urea (mg/dL)	Creatinine (mg/dL)	Glucose (mg/dL)	Troponin I (ng/mL)
Hospital Day 1	375	-	123.6	2.7	78.5	0.99	515	-
Hospital Day 2	275	12	131	2.8	70.4	0.92	378	<0.16
Hospital Day 3	156	8	135.5	3	65.1	0.89	276	-
Hospital Day 4	93.5	6.6	137.2	3.5	58.7	0.86	231	-
Hospital Day 5	54.2	6.3	138	4	54.3	0.82	198	-

## Discussion

Pneumonia as a systemic inflammatory disease

Pneumonia is increasingly recognized as a systemic inflammatory disease, extending beyond pulmonary involvement to affect multiple organ systems, including the cardiovascular system [1,2]. The inflammatory response triggered by pneumonia involves a cascade of cytokines, including IL-6, TNF-α, and IL-1β, which contribute to endothelial dysfunction and myocardial stress [2]. This inflammatory burden is particularly pronounced in older adults, who demonstrate an exaggerated cytokine response, potentially predisposing them to a higher incidence of cardiac arrhythmias [1].

AF and atrial flutter are well-documented complications of pneumonia, with recent studies estimating a 4-10% incidence of new-onset atrial arrhythmias in hospitalized pneumonia patients; however, the incidence varies significantly depending on the patient population. The risk and incidence are notably higher in critically ill patients admitted to the ICU compared to the general hospital population [3,4]. The proposed mechanisms include inflammation-induced atrial remodeling, autonomic dysfunction, and increased sympathetic drive, all of which contribute to arrhythmogenicity [2,5]. Elevated CRP levels have been strongly associated with increased atrial arrhythmia risk, suggesting that inflammation plays a pivotal role in triggering these electrical disturbances [6,9]. The presence of high CRP levels in this case aligns with existing literature, supporting the hypothesis that inflammatory stress is a key driver of arrhythmogenesis in pneumonia patients.

In addition to direct myocardial effects, systemic inflammation can promote a prothrombotic state, further increasing the risk of thromboembolic complications in pneumonia patients with new-onset atrial flutter or fibrillation [4]. Given these considerations, early identification and management of inflammation-related cardiac complications may reduce the risk of thromboembolic events, prevent hemodynamic instability, and lower ICU admission rates, ultimately improving survival and long-term cardiovascular outcomes in pneumonia patients with new-onset atrial flutter or fibrillation.

Electrolyte imbalance as a contributor to arrhythmias

Electrolyte imbalances are common in pneumonia and can further predispose patients to atrial arrhythmias. In particular, hypokalemia and hyponatremia, both present in this case, are recognized as key risk factors for atrial flutter and AF due to their effects on cardiac excitability and conduction [7,8].

Potassium plays a crucial role in stabilizing cardiac membrane potential and maintaining normal atrial conduction. Hypokalemia delays repolarization, increases atrial automaticity, and enhances reentry circuits, all of which promote atrial arrhythmogenesis [5,7]. Similarly, hyponatremia contributes to autonomic dysregulation and alters cardiac ion channel activity, further increasing susceptibility to arrhythmias [7,8].

In this case, the patient's persistent hypokalemia and hyponatremia may have exacerbated the atrial flutter, suggesting that earlier and more aggressive electrolyte correction could have mitigated arrhythmia risk. The role of diuretic therapy in worsening electrolyte imbalances should also be considered, as loop diuretics like furosemide are known to deplete potassium and sodium levels [7]. In this case, furosemide therapy was initiated to manage pulmonary congestion; however, it is essential to consider whether the patient was truly volume-overloaded or euvolemic, as diuretic-induced electrolyte disturbances can exacerbate arrhythmogenesis [7]. While hypokalemia (2.7 mmol/L on admission) was corrected with IV and oral potassium supplementation, hypomagnesemia, another key cofactor in arrhythmias, was not assessed in this patient. Magnesium depletion often coexists with hypokalemia and can contribute to arrhythmia persistence, particularly in pneumonia patients receiving diuretics [5,7,8]. This highlights the importance of monitoring electrolyte levels closely in pneumonia patients receiving diuretic therapy, particularly those at high risk for arrhythmias.

Clinical implications and learning points

Should Pneumonia Patients With Severe Inflammation Undergo Routine ECG Monitoring?

Given the well-established link between pneumonia, systemic inflammation, and atrial arrhythmias, routine ECG monitoring may be warranted in high-risk pneumonia patients, particularly those with elevated CRP, pre-existing cardiovascular disease, or electrolyte disturbances [3,5]. Early ECG screening may allow for timely detection of atrial flutter or fibrillation, enabling prompt anticoagulation and rate/rhythm control to prevent complications such as stroke and heart failure exacerbation. A CRP threshold for routine ECG screening in pneumonia patients remains an area of active investigation. Elevated CRP levels, particularly those exceeding 3 mg/L, have been associated with an increased risk of AF, suggesting that heightened inflammatory states may contribute to arrhythmogenesis [6]. However, it remains unclear whether CRP independently predicts arrhythmia onset or merely serves as a marker of systemic inflammation.

Should There be Protocols for Early Electrolyte Correction in Pneumonia Patients With Risk Factors for Arrhythmias?

Current evidence suggests that electrolyte imbalances significantly contribute to pneumonia-induced arrhythmias [7,8]. As seen in this case, hypokalemia and hyponatremia persisted despite ICU management, raising the question of whether more proactive electrolyte correction strategies could improve outcomes. Maintaining serum potassium above 4.0 mmol/L is commonly recommended to reduce arrhythmia risk, particularly in patients predisposed to atrial arrhythmias, though this target is largely extrapolated from AF management guidelines​ [5]. Future guidelines may benefit from incorporating routine potassium and sodium monitoring in pneumonia patients, with early potassium repletion to maintain levels above 4.0 mmol/L in those at high arrhythmia risk.

Do Current Guidelines Adequately Address These Risks?

While pneumonia management guidelines emphasize antibiotic therapy and respiratory support, they often underemphasize the cardiac risks associated with systemic inflammation and electrolyte imbalances [2,4]. Recognizing pneumonia as a multi-system disease rather than an isolated pulmonary infection could lead to improved clinical protocols that integrate cardiac and metabolic monitoring into pneumonia management. Current pneumonia guidelines, including those by the Infectious Diseases Society of America (IDSA) and the American Thoracic Society (ATS), primarily emphasize antimicrobial therapy and respiratory management, with limited attention to cardiovascular and metabolic risks [10]. There is increasing recognition of the need to integrate cardiac monitoring and electrolyte management into pneumonia care, as systemic inflammation and electrolyte disturbances contribute significantly to adverse cardiac outcomes ​[2,4,6,9]. Future updates to guidelines may incorporate these concerns to improve patient outcomes.

Future directions

Further research is needed to establish standardized protocols for electrolyte management and inflammation control in pneumonia patients to mitigate cardiovascular complications. To be more specific, randomized controlled trials (RCTs) are warranted to evaluate early electrolyte replacement thresholds (e.g., maintaining potassium >4.0 mmol/L) and their direct impact on arrhythmia prevention. Predictive models for arrhythmia risk stratification should be developed using machine learning algorithms incorporating CRP levels, electrolyte disturbances, and clinical severity scores to identify pneumonia patients at the highest risk of new-onset atrial flutter. Multicenter observational studies should investigate whether standardized inflammation management protocols, such as early corticosteroid use or targeted anti-inflammatory therapies, can modulate cardiovascular risk in pneumonia patients with systemic inflammation.

## Conclusions

Pneumonia extends beyond a localized pulmonary infection and should be recognized as a systemic inflammatory disease with significant cardiovascular implications. This case highlights the interplay between pneumonia-induced inflammation, electrolyte disturbances, and the development of atrial arrhythmias, reinforcing the need for comprehensive patient monitoring and management. Patients at high risk for developing atrial flutter in the setting of pneumonia include those with severe inflammation, pre-existing cardiovascular disease, ICU admission, and significant electrolyte imbalances such as hypokalemia and hyponatremia. The inflammatory burden in pneumonia, particularly in older adults, contributes to an exaggerated cytokine response that predisposes to cardiovascular complications. Additionally, the systemic inflammatory response promotes a prothrombotic state, further increasing the risk of arrhythmias and thromboembolic complications.

The assertion that early correction of electrolyte imbalances and aggressive inflammation control may reduce arrhythmia-related complications is based on known pathophysiological mechanisms linking inflammation and atrial fibrillation risk. Elevated CRP levels and electrolyte disturbances can be associated with atrial arrhythmogenesis, but direct clinical evidence proving that proactive correction prevents new-onset atrial flutter remains limited. Proactive electrolyte management should aim to maintain potassium levels above 4.0 mmol/L in at-risk patients, as hypokalemia facilitates atrial arrhythmias by delaying repolarization and increasing reentry circuits. However, unnecessary supplementation should be avoided to prevent hyperkalemia, which carries independent arrhythmic risks, particularly in patients with impaired renal function. Likewise, careful correction of hyponatremia is essential to avoid neurological complications. Recognizing pneumonia as a multi-system disease rather than an isolated respiratory infection may lead to improved clinical guidelines and patient outcomes by integrating cardiac monitoring and metabolic optimization into standard pneumonia care.
